# Sustained Retinal Defocus Increases the Effect of Induced Myopia on the Retinal Astrocyte Template

**DOI:** 10.3390/cells13070595

**Published:** 2024-03-29

**Authors:** Carol R. Lin, Abduqodir Toychiev, Reynolds K. Ablordeppey, Miduturu Srinivas, Alexandra Benavente-Perez

**Affiliations:** Department of Biological Sciences, State University of New York College of Optometry, New York, NY 10036, USA; clin@sunyopt.edu (C.R.L.); a5toychiev@gmail.com (A.T.); rablordeppey@sunyopt.edu (R.K.A.); msrinivas@sunyopt.edu (M.S.)

**Keywords:** myopia, astrocytes, aging, neurovascular unit, marmoset

## Abstract

The aim of this article is to describe sustained myopic eye growth’s effect on astrocyte cellular distribution and its association with inner retinal layer thicknesses. Astrocyte density and distribution, retinal nerve fiber layer (RNFL), ganglion cell layer, and inner plexiform layer (IPL) thicknesses were assessed using immunochemistry and spectral-domain optical coherence tomography on seventeen common marmoset retinas (*Callithrix jacchus*): six induced with myopia from 2 to 6 months of age (6-month-old myopes), three induced with myopia from 2 to 12 months of age (12-month-old myopes), five age-matched 6-month-old controls, and three age-matched 12-month-old controls. Untreated marmoset eyes grew normally, and both RNFL and IPL thicknesses did not change with age, with astrocyte numbers correlating to RNFL and IPL thicknesses in both control age groups. Myopic marmosets did not follow this trend and, instead, exhibited decreased astrocyte density, increased GFAP+ spatial coverage, and thinner RNFL and IPL, all of which worsened over time. Myopic changes in astrocyte density, GFAP+ spatial coverage and inner retinal layer thicknesses suggest astrocyte template reorganization during myopia development and progression which increased over time. Whether or not these changes are constructive or destructive to the retina still remains to be assessed.

## 1. Introduction

Myopia is a refractive condition that can cause retinal pathology at severe stages. It is a preeminent risk factor for developing retinopathies such as choroidal neovascularization, glaucoma, and other maculopathies [1,2]. The projected global increase in myopia prevalence is staggering, predicted to affect almost 5 billion people by the year 2050 [3,4], and will likely lead to public health crises and both financial and community burdens on healthcare systems worldwide. Despite this increase in myopia prevalence, the mechanisms underlying myopic degeneration and its associated retinopathies remain unknown [3]. There are no known strategies for preventing disease progression, and no early-stage diagnostic markers of myopic pathology [1,2].

Myopia of all degrees is associated with higher risk for glaucoma [5], chorioretinopathy [6], cataracts [7], and retinal detachment [8]. The development and progression of myopia also results in structural changes, including inner retinal, choroidal, and scleral thinning [9,10,11] and reduced retinal pigment epithelium cell density [10,12,13]. Both the sclera and the choroid are affected by myopic stretch [14,15], with genetic and cellular changes suggesting the existence of differential changes in mechanical response genes [16]. Cellular changes to the inner retina due to myopia progression have also been identified. Previous work from our lab has described a decrease in astrocyte density along with increased glial fibrillary acidic protein (GFAP) spatial coverage in myopic marmoset eyes (*Callithrix jacchus*), an established non-human primate model of myopia. This has also been associated with retinal nerve fiber layer (RNFL) thickness changes [17]. There was also decreased peripheral retinal branchpoint density, along with a central increase in string vessels and retinal branchpoint density [17]. Supporting the existence of retinal glia alterations in myopia, increased GFAP expression and both astrocyte and Müller cell morphology changes have been demonstrated in a mouse model of myopia [18].

The relationship between neurons, glia, and the vasculature within the retina is collectively called the retinal neurovascular unit, and is essential to maintaining homeostasis and modulating neuronal activities [19]. Functions of the neurovascular unit include structure regulation and nutritional support [19,20], blood–retinal barrier integrity maintenance [21], and debris phagocytosis [20]. The neurovascular unit, upon the onset of systemic pathology, wields a biphasic influence: degenerative when acute, and regenerative when chronic [22]. All glial, vascular, and neuronal cells in the neurovascular unit work together in a feedback loop under both normal and abnormal development [23,24]. Astrocytes, located centrally between endothelial cells of the vasculature and neurons [25], are a crucial element of the neurovascular unit and cleverly adapt and help to regulate neuronal synaptic activity and metabolism [26,27]. Astrocytes are highly heterogeneous and are among the most commonly found glia cell type within the retina [28], and their degeneration and reactivity precede ganglion cell degeneration and pathological neovascularization [23,29], possibly leading to the progression of ocular diseases like glaucoma or oxygen-induced retinopathy. 

Experimental research is crucial to bridge etiological and mechanistic knowledge gaps with the end goal to advance human treatment [30,31,32]. The common marmoset is an exceptional non-human primate (NHP) model for vision and neuroscience research due to the presence of a high optical quality foveated eye akin to the human eye, quick development, small animal size, and ease of care and handling [33,34,35]. In our lab, common marmosets are successfully induced with varying degrees of myopia utilizing negative-powered contact lenses, following a lens-induced myopia paradigm [33,34,35,36,37]. There is no other lens-induced myopia NHP model, with a directly comparable ocular structure and physiology to that of the human eye. Despite great progress in the research fields of myopia control and etiology, myopia’s longitudinal effect on the retinal astrocyte template and RNFL thickness, and how they change with longer periods of induced myopia, remains unknown. In this study, the astrocyte density and distribution as well as RNFL, ganglion cell layer (GCL), and inner plexiform layer (IPL) thicknesses were assessed in an experimental NHP myopia model to study progressive myopia’s effect on the ocular tissues. 

## 2. Methods

### 2.1. The Myopia Marmoset Model

Seventeen marmoset eyes were included, grouped into treated or age-matched untreated controls: six 6-month-old myopic eyes (6 mM) induced with myopia for 4 months, five age-matched 6-month-old untreated controls (6 mC), three 12-month-old myopes induced with myopia for 10 months (12 mM) and three age-matched 12-month-old controls (12 mC). Myopes were induced with myopia using soft negative-powered full-field single-vision contact lenses (either -5D or -10D powers) [17] for 4 months from 2 to 6 months of age (6-month-old myopic marmosets, 6 mM), or for 10 months from 2 to 12 months of age (12-month-old myopic marmosets, 12 mM). Details of each animal included (age, axial length, refractive error, and gender) can be found in Table 1. Statistical power analysis using published data from our lab indicates that 4 eyes per experimental group in our younger cohort provides 80% power for our statistical analysis [17]. Our data are less variable in the older marmosets induced with sustained myopia for longer, and the statistical power analysis indicates that 3 eyes per older control and older myopic group provides 80% power for our statistical analysis. All animal care and experimental protocols were approved by the State University of New York College of Optometry Institutional Animal Care and Use Committee (IACUC), and performed as recommended by the US National Research Council’s Guide for the Care and Use of Laboratory Animals, the ARVO statement for the use of animals in ophthalmic and vision research, the Guide for the Care and Use of Laboratory animals, and the US Public Health Service’s Policy on Humane Care and Use of Laboratory Animals.

Cycloplegic refractive error was measured with the Nidek ARK-900 autorefractor (Nidek Co., LTD, Gamagori, Japan) on alert marmosets. Cyclopleged animals were subsequently anesthetized (alphaxalone, 15 mg/kg, intramuscular) for axial length (AL) and optical coherence tomography (OCT) measurements. AL was performed using an ultrasound biometer (A-scan ultrasound 25 MHz, Panametrics, NDT Ltd., Waltham, MA, USA), with eight individual A-scan traces averaged and taken to be the axial length of each eye measured. RNFL, GCL, and IPL thicknesses were then measured using a Spectral Domain Optical Coherence Tomography machine (SD-OCT) (Bioptigen SD-OCT; 12 × 12 mm^2^, 700 A-scans/B-scan × 70 B-Scans × 5 Frames/B-scan, Bioptigen Inc., Durham, NC, USA). Marmosets wore custom-made plano rigid gas permeable lenses (3.75 mm base curve/5 mm diameter, 0 D; Conforma Laboratories, Inc., Norfolk, VA, USA) for the duration of OCT measurements to prevent ocular surface tear film evaporation. Cycloplegic refractive error, axial length, and optical coherence tomography were performed at baseline and at the end of treatment before collection of retinal tissue. SD-OCT images were segmented using The Iowa Reference Algorithms (Version 3.6, Iowa Institute for Biomedical Imaging, Iowa City, IA, USA). RNFL, GCL, and IPL exhibit retinal thinning and cellular template remodeling secondary to myopia development [9,17] in marmoset eyes, which is why the inner retinal layer thicknesses were analyzed.

### 2.2. Collection of Retinal Tissue and Immunohistochemical Staining of Retinal Flatmounts

Eyes were enucleated and washed with phosphate-buffered saline (PBS; ThermoFisher, Waltham, MA, USA) at the end of treatment. Retinas were fixed in Paraformaldehyde (PFA) 4% (Santa Cruz Biotechnology, Dallas, TX, USA) for 30 min, then washed with PBS five times for 30 min each, and incubated in blocking buffer (5% normal donkey serum (Sigma Aldrich, St. Louis, MO, USA), 0.5% Triton X (Sigma Aldrich, St. Louis, MO, USA), and PBS) for one hour. After blocking, retinal tissue was incubated for 3 days with primary antibodies in blocking buffer at 4 °C. The primary antibodies used were mouse anti-GFAP to detect GFAP intermediate filament protein (1:500, Catalog #MAB360, Millipore Sigma, Burlington, MA, USA) and rabbit anti-Sox 9 to detect astrocyte nuclei (1:1000, Catalog #AB5535, Sigma Aldrich, St. Louis, MO, USA). After primary antibody incubation period, retinas were washed with PBS six times for 10 min each and incubated with secondary antibodies (donkey-anti mouse 594 antibody (1:200, ThermoFisher, Catalog #A21203, Waltham, MA, USA) and donkey-anti rabbit 488 (1:200, Catalog #A32790, ThermoFisher, Waltham, MA, USA)) for 2 days. Retinal tissue was then washed one time for 30 min, and six times for 10 min each time with PBS. Retinas were inspected for debris or tissue abnormalities prior to plating onto SuperFrost slides (ThermoFisher, Waltham, MA, USA). Cover slips were then placed on objectives with DAPI mounting medium (SKU: H-1200-10, Vector Laboratories, Newark, CA, USA) and stored at −20 °C.

### 2.3. Confocal Microscopy

Plated retinal samples underwent confocal microscopy with the Olympus FV1200 MPE confocal microscope (Olympus Corporation, Tokyo, Japan). Images were gathered and analyzed by one blind investigator in a randomized order. Both eyes were kept separately after enucleation and distinguished by noting the presence of the foveal pit and temporal region. Twelve images (317 μm × 317 μm horizontally, and 10 μm deep vertically) were taken from each of the seventeen retinas quantified. Multiplane z-series were taken using the 40× magnification objective, with each section 1 μm in depth. The confocal microscope then processed each stack of 10 sections to form single z-stacks of images from the retinal tissue. Fiji software (Mac OS, version 20200928-2004) was used to process images, to quantify astrocyte density (astrocytes nuclei per mm^2^) and GFAP immunopositive spatial coverage (percentage of GFAP immunopositive staining per mm^2^). The two parameters were assessed via retinal quadrant imaging (nasal, temporal, inferior, and superior) of different retinal locations (parafoveal, peripapillary, and peripheral). The temporal region contained the fovea, while the nasal region was directly opposite the temporal region. Inferior and superior retinas were categorized depending on the eye. Quadrantal and regional analysis of the retina were performed to help identify whether local changes occurred in myopic eyes known to experience asymmetrical growth.

### 2.4. Image and Statistical Analysis

Astrocyte density was calculated using the Fiji cell counter function, by manually counting the number of astrocyte nuclei in every image and converting to astrocytes/mm^2^. The image was then split into separate color channels to identify the channel corresponding to GFAP-positive staining (red). The red channel’s image was made “binary”, (Process, Binary, Make Binary) then converted to mask (Process, Binary, Convert to Mask). The “masked” image is a white background with black particles, with the black particles subsequently quantified as “percentage area covered” (Analyze, Analyze Particles, Ok). We interpreted this automated Fiji value as GFAP-immunopositive staining and astrocyte spatial coverage. Averaged values of the central RNFL, GCL, and IPL in both 6- and 12-month-old marmosets were gathered. Previous studies have confirmed the ability and validity of SD-OCT scans to be used for measuring marmoset retinal thickness [9,17].

A two-dimensional magnification correction was also performed in order to account for myopic retinal growth using a tangential equation. Data were assessed for normality and analyzed using one-way analysis of variance (ANOVA) with post hoc analysis using Tukey tests at significance level α = 0.05, and Student’s *t*-test was used to assess any differences between different age groups of control and myopic eyes. Graphs were made using OriginPro 2023 software (OriginLab, Northampton, MA, USA), and figures were assembled in Adobe Indesign (Adobe, San Jose, CA, USA).

## 3. Results

After correcting for magnification, the average myopic retinal area was similar to that of the control eyes (0.64 mm controls vs. 0.65 mm in myopes), confirming the minimal effect of image magnification on cell density and distribution quantifications.

**Figure 1:**
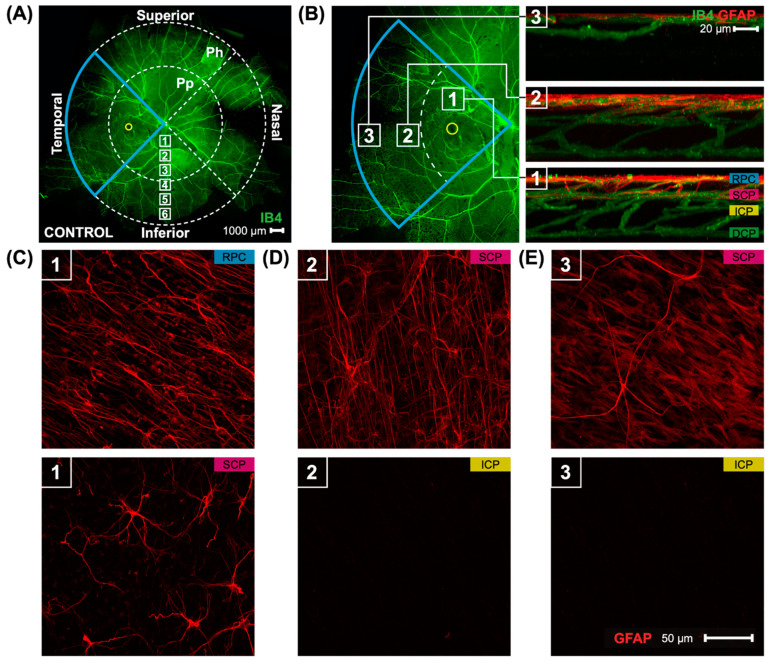
A whole marmoset retina’s superficial vasculature is seen, along with images of the astrocytes and astrocyte layers in the three retinal regions studied. The figure is modified from Lin et al. [17]. (**A**) A control marmoset’s retinal vasculature map [green; (ID: C16 Left)] is shown. Outlined in blue is the temporal region of the eye, with fovea location denoted by a yellow circle. The retinal vasculature shown is visualized using conjugated Isolectin-488, and consists of multiple images acquired at 4× magnification and stitched together using Photoshop. The peripapillary (Pp) and peripheral regions (Ph) quantified in this study are shown, as are the locations where the images were taken. Focal areas away from the center of the retina (optic nerve) to periphery are shown via the white boxes numbered 1–6. Inferior, superior, temporal, and nasal quadrants of the retina are also shown. Scale bar, 1000 μm. (**B**) An image of the temporal retina (**left**) is highlighted in blue, and visualized using isolectin (green). The fovea location is shown by the yellow circle. Numbers in the white boxes represent areas evaluated (1: parafoveal, 2: peripapillary, 3: periphery). Reconstructed images (**right**) from areas 1, 2, and 3 show the distribution of astrocytes (red) and the vasculature (green). The four vascular plexi, from the inner to outer retina, are the radial peripapillary capillary (RPC), superficial (SCP), intermediate (ICP), and deep (DCP) plexi. Scale bar, 20 µm. (**C**–**E**) Images of representative retinal astrocytes (red) found in areas 1, 2, and 3. Scale bar, 50 µm. In area 1 (parafovea), astrocytes are distributed among two vascular layers, the RPC and SCP layers. In other areas (2, 3), astrocytes are found only in one layer, the superficial layer.

Marmoset retinas were separated into four quadrants: superior, inferior, nasal, and temporal quadrants (Figure 1A) (scale bar 1000 μm). The presence of the foveal pit (yellow circle) demonstrated which quadrant was the temporal quadrant (blue wedge outline). Each quadrant was then split into two regions: peripapillary (Pp) and periphery (Ph). Confocal images in the peripapillary and peripheral retina in this study were taken at 40× magnification, with six focal areas of 40× magnification present when moving in a straight line from the optic nerve head to the peripheral retina (white boxes, focal areas 1–6). Images quantified in this study were taken in all four quadrants in focal area 3 for the peripapillary quantification and focal area 6 for the periphery quantification, and were also directly adjacent to the foveal pit (yellow circle) for a total of 12 images per retina. Locations of the parafoveal (1), peripapillary (2), and peripheral (3) regions may be seen (Figure 1B) alongside 3D reconstructed images of the vasculature and astrocytes in the three areas. Marmoset retinas contain two layers of parafoveal astrocytes (Figure 1B, box 1), both located in the RNFL: the more superficial layer lies closer to the inner limiting membrane, and the deeper layer lies closer to the GCL. The two layers of astrocytes correspond to the two most superficial vascular plexi, the radial peripapillary capillary plexus (RPC), and the superficial capillary plexus (SCP) [17]. The deeper vascular layers of the primate retina, the intermediate capillary plexus (ICP) and deep capillary plexus (DCP), do not contain astrocytes. The peripapillary and peripheral regions of marmoset retinas have one layer of astrocytes only (Figure 1B boxes 2 and 3, respectively). Representative images are shown in Figure 1C–E of the two layers of parafoveal astrocytes (Figure 1C), the single layer of peripapillary astrocytes (Figure 1D), and the single layer of peripheral astrocytes (Figure 1E). Astrocyte morphology and distribution in marmoset eyes has been described in previous work from our lab [17]. 

### 3.1. Imposing Negative Defocus for 10 Months vs. 4 Months Increases the Effect of Myopia on Retinal Astrocyte Density and GFAP Immunopositive Spatial Coverage

**Figure 2:**
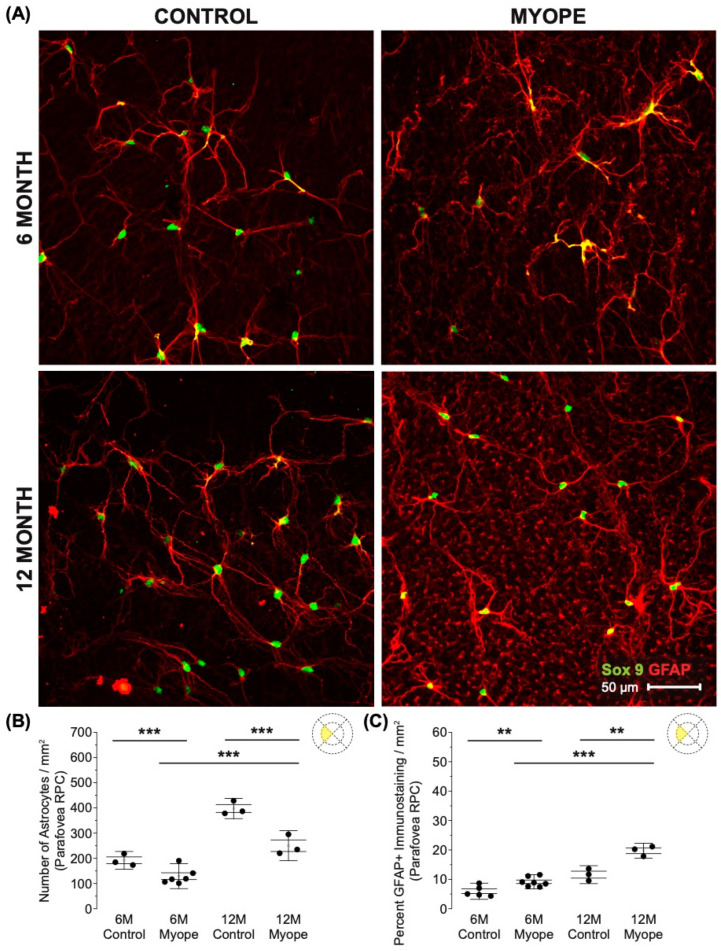
Myopic marmoset retinas exhibit decreased astrocyte density and increased GFAP+ spatial coverage in the parafoveal RPC layer retina. ** = *p* < 0.01, *** = *p* < 0.001. (**A**) Some representative images of control marmoset parafoveal RPC layer astrocytes (6-month control ID tag: H16 Right, 12-month control ID tag: X15 Right) and myopic marmoset parafoveal RPC layer astrocytes (6-month myope ID tag: P17 Right, 12-month myope ID tag: I19 Right). Astrocyte cell nuclei and spatial distribution were labeled with Sox9 (green) and GFAP (red) markers, respectively. (**B**) Analysis was performed for astrocyte density in the parafoveal RPC layer (6-month control *n* = 5, 6-month myopic *n* = 6, 12-month control *n* = 3, 12-month myopic *n* = 3). Data are shown as a box plot with SE as the box and SD for whiskers, with the yellow section in the pie chart indicating the region that was analyzed (parafoveal RPC layer) in (**B**,**C**). Significantly decreased parafoveal RPC layer astrocyte density was seen in both young and older myopic eyes (6-month *p* < 0.001; 12-month *p* < 0.001). (**C**) Increased GFAP+ spatial coverage was seen in the myopic parafoveal RPC layer of astrocytes (6-month *p* < 0.01), which was still significant in aged myopic retinas (12-month *p* < 0.01).

Astrocyte density was lower in the RPC parafoveal layer of myopic eyes (Figure 2A,B; 6 m *p* < 0.001, 12 m *p* < 0.001), with increased GFAP+ spatial coverage noted in myopic eyes compared to that of controls (Figure 2C: 6 m *p* < 0.01, 12 m *p* < 0.01). The GFAP+ spatial coverage may have originated from either or both astrocytes and Müller glia cells, with myopic animals treated for a longer duration of time showing a greater effect than younger myopic animals (Figure 2B: *p* < 0.001, Figure 2C: *p* < 0.001).

**Figure 3:**
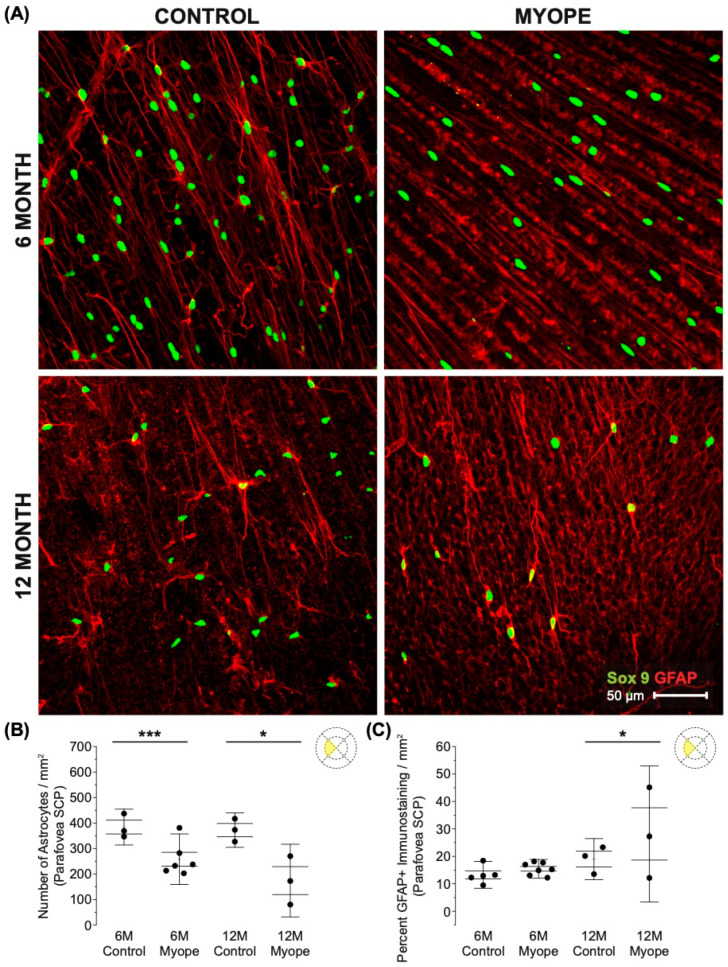
Myopic marmoset retinas exhibit decreased astrocyte density and increased GFAP+ spatial coverage in the parafoveal SCP layer retina. * = *p* < 0.05, *** = *p* < 0.001. (**A**) Some representative images of control marmoset parafoveal SCP layer astrocytes (6-month control ID tag: H16 Right, 12-month control ID tag: S15 Right) and myopic marmoset parafoveal RPC layer astrocytes (6-month myope ID tag: O17 Right, 12-month myope ID tag: I19 Right) are shown. Astrocyte cell nuclei and spatial distribution were labeled with Sox9 (green) and GFAP (red) markers, respectively. (**B**) Analysis was performed for astrocyte density in the parafoveal SCP layer (6-month control *n* = 5, 6-month myopic *n* = 6, 12-month control *n* = 3, 12-month myopic *n* = 3). Data are shown as a box plot with SE as the box and SD for whiskers, with the yellow section in the pie chart indicating the region that was analyzed (parafoveal SCP layer) in (**B**,**C**). Significantly decreased parafoveal SCP astrocyte density was identified in both young and older myopic eyes (6-month *p* < 0.001; 12-month *p* < 0.05). (**C**) Increased GFAP+ spatial coverage was seen in the older myopic parafoveal SCP layer of astrocytes (6-month *p* > 0.05, 12-month *p* < 0.05).

Astrocyte density was lower in the SCP parafoveal layer of myopic eyes (Figure 3A,B: 6 m *p* < 0.001, 12 m *p* < 0.05), and also demonstrated increased GFAP+ spatial coverage in myopic eyes compared to controls (Figure 3C: 6 m *p* > 0.05, 12 m *p* < 0.05). The GFAP+ spatial coverage may have originated from either or both astrocytes and Müller cells, with myopic animals treated for a longer duration of time showing a greater effect than younger myopic animals (Figure 3B: *p* < 0.001, Figure 3C: *p* < 0.001).

**Figure 4:**
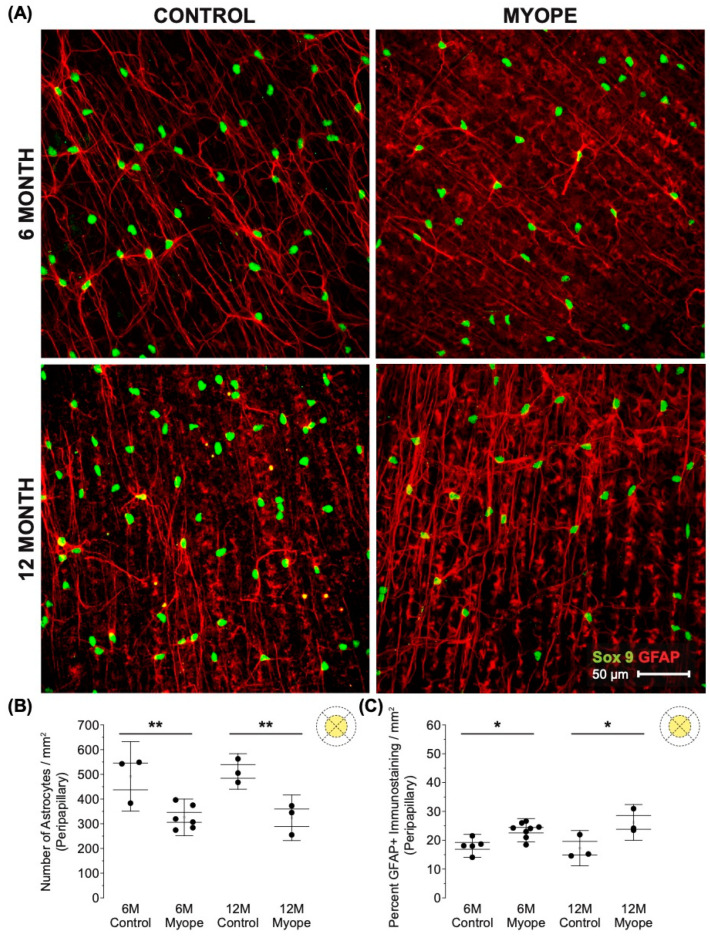
Myopic marmoset retinas exhibit decreased astrocyte density and increased GFAP+ spatial coverage in the peripapillary retina. * = *p* < 0.05, ** = *p* < 0.01. (**A**) Some representative images of control marmoset peripapillary astrocytes (6-month control ID tag: H16 Right, 12-month control ID tag: X15 Right) and myopic marmoset peripapillary astrocytes (6-month myope ID tag: O17 Right, 12-month myope ID tag: I19 Right) are shown. Astrocyte cell nuclei and spatial distribution were labeled with Sox9 (green) and GFAP (red) markers, respectively. (**B**) Analysis was performed for astrocyte density in the peripapillary region (6-month control *n* = 5, 6-month myopic *n* = 6, 12-month control *n* = 3, 12-month myopic *n* = 3). Data are shown as a box plot with SE as the box and SD for whiskers, with the yellow section in the pie chart indicating the region that was analyzed (peripapillary) in (**B**,**C**). Significantly decreased peripapillary astrocyte density was seen in both young and older myopic eyes (6-month *p* < 0.01; 12-month *p* < 0.01). (**C**) Increased GFAP+ spatial coverage was seen in the myopic peripapillary retina (6-month *p* < 0.05, 12-month *p* < 0.05).

Decreased astrocyte density was noted in the peripapillary region of myopic eyes (Figure 4A,B; 6 m *p* < 0.01, 12 m *p* < 0.01), and also showed increased GFAP+ spatial coverage in myopic eyes compared to controls (Figure 4C: 6 m *p* < 0.05, 12 m *p* < 0.05). GFAP+ spatial coverage may have originated from either or both astrocytes and Müller cells, with myopic animals treated for a longer duration of time showing a greater effect than younger myopic animals (Figure 4B: *p* < 0.001, Figure 4C: *p* < 0.001).

**Figure 5:**
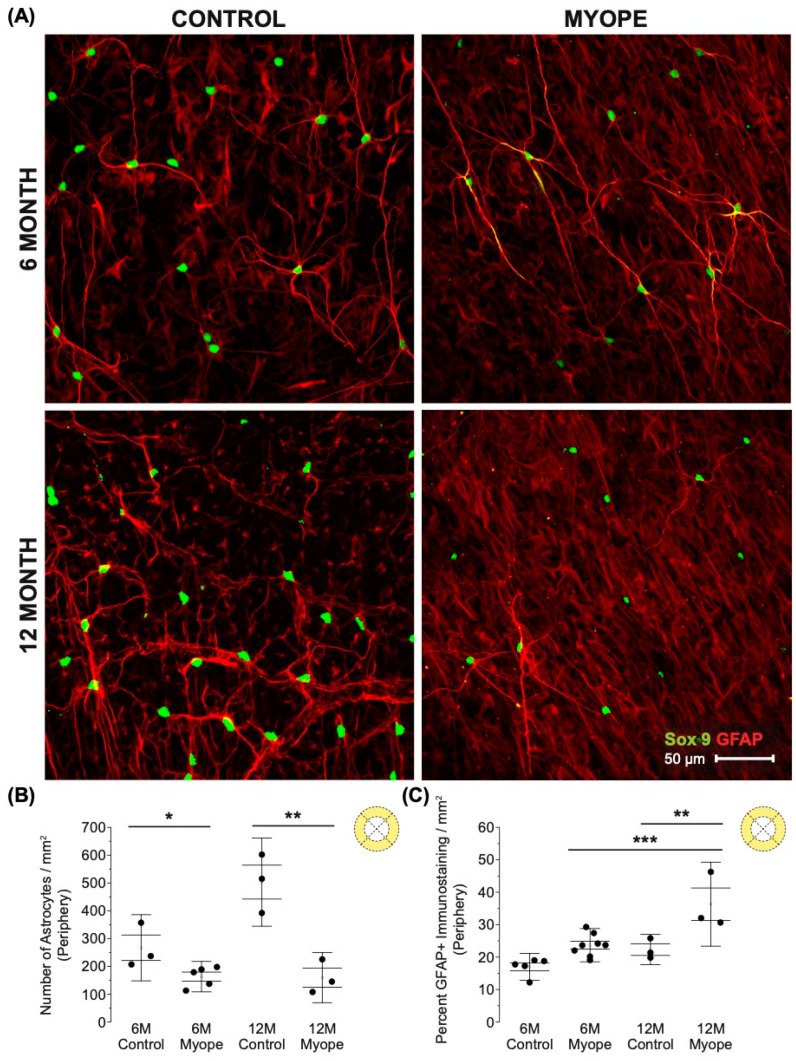
Myopic marmoset retinas exhibit decreased astrocyte density and increased GFAP+ spatial coverage in the peripheral retina. * = *p* < 0.05, ** = *p* < 0.01, *** = *p* < 0.001. (**A**) Some representative images of control marmoset peripheral astrocytes (6-month control ID tag: H16 Right, 12-month control ID tag: X15 Right) and myopic marmoset peripheral astrocytes (6-month myope ID tag: P17 Right, 12-month myope ID tag: I19 Right) are shown. Astrocyte cell nuclei and spatial distribution were labeled with Sox9 (green) and GFAP (red) markers, respectively. (**B**) Analysis was performed for astrocyte density in the peripheral region (6-month control *n* = 5, 6-month myopic *n* = 6, 12-month control *n* = 3, 12-month myopic *n* = 3). Data are shown as a box plot with SE as the box and SD for whiskers, with the yellow section in the pie chart indicating the region that was analyzed (peripheral) in (**B**,**C**). Significantly decreased peripheral astrocyte density was identified in both young and older myopic eyes (6-month *p* < 0.05; 12-month *p* < 0.01). (**C**) Increased GFAP+ spatial coverage was seen in older myopic peripheral retinas (6-month *p* > 0.05, 12-month *p* < 0.01).

Decreased astrocyte density was noted in the peripheral retina of myopic eyes (Figure 5A,B: 6 m *p* < 0.05, 12 m *p* < 0.01), and demonstrated increased GFAP+ spatial coverage in myopic eyes compared to that of controls (Figure 5C: 6 m *p* > 0.05, 12 m *p* < 0.01). The GFAP+ spatial coverage may have originated from either or both astrocyte and Müller cells, with myopic animals treated for a longer duration of time showing a greater effect than younger myopic animals (Figure 5B: *p* < 0.001, Figure 5C: *p* < 0.01).

### 3.2. Eyes Induced with Myopia for 10 Months Had Thinner Retinal Nerve Fiber Layer (RNFL) and Inner Plexiform Layer (IPL) Thicknesses than Eyes Induced with Myopia for 4 Months and Controls

**Figure 6:**
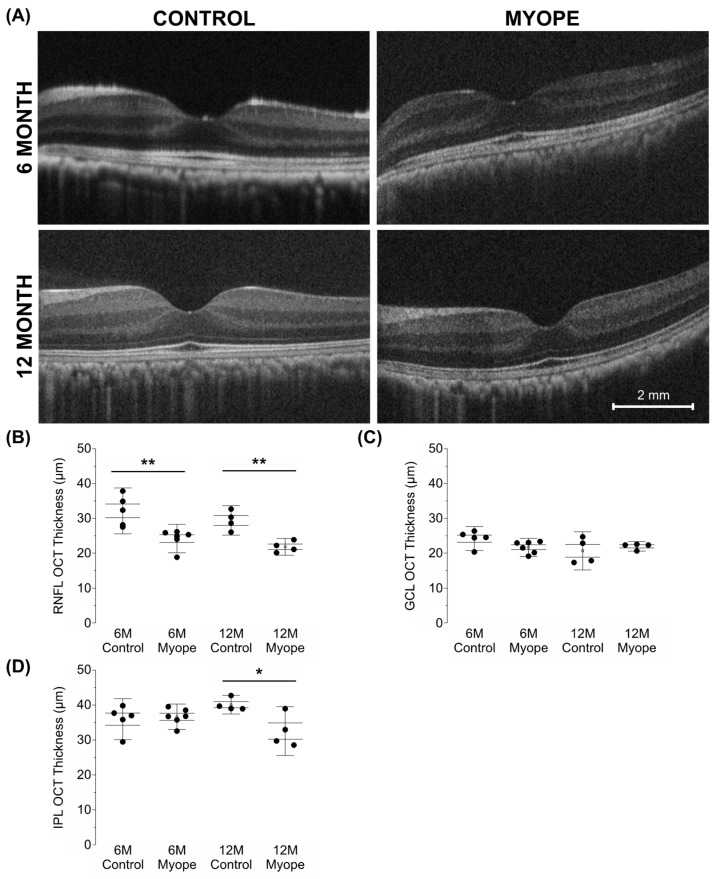
Myopic marmosets have thinner RNFL and IPL thicknesses, with no change to GCL thickness. * *p* < 0.05, ** *p* < 0.01. (**A**) Representative images of macular OCTs gathered from a 6-month-old control (**top left**), myope treated with negative lenses for four months (**top right**), 12-month-old control (**bottom left**), and myope treated with negative lenses for 10 months (**bottom right**). (**B**) Myopic RNFL was significantly thinner parafoveally, with effects exacerbated by age (6-month *p* < 0.01, 12-month *p* < 0.01). (**C**) The parafoveal myopic GCL thickness did not differ from that of the control parafoveal GCL thickness (6-month *p* > 0.05, 12-month *p* > 0.05). (**D**) The parafoveal IPL of older myopes was significantly thinner than that of age-matched controls (6-month *p* > 0.05, 12-month *p* < 0.05).

In myopic eyes, the parafoveal RNFL was significantly thinner compared to that of the age-matched controls and did not change between myopic eyes induced for 4 versus 10 months (Figure 6A: 6 m *p* < 0.01, 12 m *p* < 0.01). There was no difference in GCL thickness between treatment groups (Figure 6B: 6 m *p* > 0.05, 6 m *p* > 0.05). However, the inner plexiform layer (IPL) of myopic eyes treated for longer was thinner than that of the age-matched controls (Figure 6C: 6 m *p* > 0.05, 12 m *p* < 0.05). These changes were associated with increased axial length, decreased astrocyte density, and increased GFAP+ spatial coverage, with the decreases in RNFL and IPL thicknesses in myopic eyes induced for 10 months notable even after myopic magnification correction.

## 4. Discussion

This study provides evidence that sustained exposure to negative defocus and myopia development exacerbates the changes known to occur in astrocytes and inner retinal thicknesses in a lens-induced myopia NHP model. Myopic marmosets had lower astrocyte density, increased GFAP+ spatial coverage pan-retinally, and thinner RNFLs and IPLs compared to age-matched controls. Myopia’s effect on the astrocyte, GFAP+ template, and inner retinal thicknesses was greater in marmosets induced with myopia for 10 months compared to those induced for 4 months. 

Astrocytes have become an important subject of retina research due to their critical role in neuronal support as fundamental players in the metabolism and homeostasis of the neurovascular unit [23,38,39,40]. They have been studied in multiple animal species, including mammals [17,41,42,43,44,45], mice [41,46,47], cats [42], and humans [48,49,50]. In primates and mice, retinal astrocytes are radial in shape as they exit the optic nerve, and stellate towards the periphery [40,45,50,51]. Their numbers are proportional to RNFL thickness, with the most astrocytes found at the optic nerve [44]. Astrocytes produce many factors such as Inter-leukin 33 [52] (protein signaling tissue damage and promoting homeostatic tissue development and remodeling), ZO-1 [53] (tight-junction-associated protein crucial for blood–retinal barrier regulation), TNF-α [54] (pro-inflammatory cytokine affecting macrophage activity and regulating other pro-inflammatory cytokines), and astroglial NF-Κb [55] (transcription factor regulating cellular behaviors like inflammatory responses, cellular growth and apoptosis) that target and modulate retinal ganglion cells [52,56]. Therefore, any potential alterations to the retinal astrocyte template during myopia development and progression may also have an effect on retinal ganglion cells.

### 4.1. The Decrease in Parafoveal Astrocyte Density and Associated Increased GFAP+ Spatial Coverage Is Greater in Marmosets Induced with Myopia for 10 Months vs. 4 Months

In the parafovea of myopic marmosets, there is a time-dependent decrease in astrocyte density that affects the astrocytes within the superficial capillary plexus (SCP) more than those located in the radial peripapillary capillary plexus (RPC). The decrease in astrocytes in the parafovea concurrently shows an increase in GFAP spatial coverage that is significantly greater in the older myopes that have experienced myopia for longer. These results suggest an astrocyte template reorganization in the central retina as a consequence of one year of sustained myopic growth. The fovea and parafoveal regions are often the most severely affected areas in disorders causing retinal traction such as high myopia and diabetes [57], in part due to the higher metabolic activity associated with the high neuronal density in the area [43,58,59,60]. Astrocytes and Muller cells function together as a viscoelastic network that mediates foveal structure stability [57] and mechanical tissue homeostasis [61,62,63]. The decrease in parafoveal astrocyte density and parallel increase in GFAP spatial coverage suggests that myopic eye growth has a more significant effect on central retinal glia when eyes have experienced sustained myopic growth for longer periods of time and are older. Mechanical stress is necessary in developing retinal circuitry in normal development [64]. However, when mechanical stressors act on the retina for long periods of time (like in myopic eye growth or after increased eye pressure), mechanosensitive ion channels may get activated [65] and can lead to alterations in axonal transport that may result in the biochemical dysregulation of critical neurotrophic factors crucial for ganglion cell survival [66,67,68,69,70]. The health of Müller, astrocyte, and ganglion cells is critical for axon survival and regeneration [29], and these cells are also extremely important for mechanosensitivity regulation [71], a hallmark feature of myopic stretch. There is evidence of increased GFAP immunoreactivity in human retinas as they age [72,73,74,75,76], which is also accompanied by a decrease in astroglial cell density [77,78,79,80]. These GFAP expression changes can compromise retinal ganglion cells’ glial support and increase the risk of ganglion cell axonal damage and dysfunction. Ocular diseases with increased GFAP expression include macular degeneration [80], Alzheimer’s disease [8], multiple sclerosis [81], and retinal degeneration [82,83]. Interestingly, mice induced with form deprivation myopia [18] also exhibit an increased GFAP reactivity that may translate into increased foveal vulnerability and subsequent changes to central retinal health in progressive myopia. Some retinal conditions commonly afflicting the posterior pole of degenerative myopes include myopic foveal retinoschisis [84], lacquer cracks [85], Fuch’s spots [86], and staphylomas [87]. The findings in this study might represent subclinical cellular changes affecting glial cells and preceding the presence of myopic retinal changes. 

### 4.2. The Decrease in Peripapillary and Peripheral Astrocyte Density and Associated Increased GFAP+ Spatial Coverage Is Greater in Marmosets Induced with Myopia for 10 Months vs. 4 Months

In comparison to age-matched controls and myopic marmosets treated for a shorter period of time, older myopic marmosets treated for longer demonstrate decreased astrocyte density and increased GFAP+ spatial coverage in the peripapillary and peripheral retinas. The optic nerve head and its surroundings are crucial to the adequate functioning of all cells comprising the neurovascular unit [19]. In particular, the peripapillary region is especially important to progressive diseases like glaucoma [88], diabetes [89], and optic nerve edema [90]. This region’s retinal ganglion cells are most likely to be damaged during the onset of glaucoma [91,92], in part because it contains a gap in the corneo-scleral shell [93], which typically gives rise to a concentration in stress or strain [94]. Recently, mechanosensitivity has been shown to be a feature of many soft tissue cell types [95,96,97], including retinal ganglion cells [71,98,99] and astrocytes [100]. Glial cells are a part of the cascade of pathological neuron damage that comes after ischemic or mechanical insults [101]. Ganglion cells contain metabolically expensive axons, making them particularly vulnerable to disruptions in nutrition and signaling molecules, which are provided by cells like glial and endothelial cells [71]. Mechanical stress, especially in conditions characterized by sustained mechanical stretch such as myopic growth, may especially cause duress in supporting sensory systems like somatosensation and vision [102,103]. In addition, distinct astrocytic morphological changes, astrocyte hypertrophy, and increased GFAP expression have been shown in human and rat retinal tissue with aging [80,104,105]. Aging is also associated with decreased astrocyte density in human [80] and rat retinas [106]. In addition, aging plays an important role in pathologic optic nerve head remodeling [107]. Therefore, we hypothesize that myopia’s effect on the retinal astrocyte template interacts with age and increases the risk for mechanical insult to ganglion cells due to the remodeling occurring near or at the optic nerve.

Eyes with high myopia have increased prevalence of optic nerve damage [108] which is possibly related to the morphological consequences of myopic stretch. An upregulation of GFAP in optic nerve [39,109,110,111] and retinal astrocytes [101] has been described in human glaucoma. While optic nerve astrocytes may not perform the same functions as astrocytes in the retina, especially when experiencing increased reactivity, in normal and disease states they share multiple common properties [112]. Optic nerve astrocytes are very important for supporting lamina structure, are transversely oriented to ganglion cell axons [92], and are also known to directly sense and react to mechanical stimuli [100]. With reactivity, optic nerve head astrocytes increase their plasticity and can undergo reversible reactive remodeling in cases of subtle, transient injury [113]. On the contrary, when retinal astrocytes become reactive, they experience more persistent structural and functional changes than optic nerve head astrocytes, including an increase in GFAP and soma diameter shrinkage [114]. Reactivity also seems to occur in microdomains, and areas of decreased astrocyte density may coincide with areas of increased GFAP [114]. Both optic nerve and retinal astrocyte reactivities are significantly increased with increased severity and stress duration [114].

The significantly greater astrocytic changes found in myopic marmosets induced with myopia for longer periods might be associated with thinner RNFL [17]. Peripheral retinas of myopic eyes tend to be thinner and can exhibit myopia-related peripheral retinal degenerations, like lattice degeneration, retinal holes/detachments and paving stone degeneration [115]. As myopic eyes elongate, glial cells may also experience the stretch associated with increased myopic growth. Whether the myopic decrease in astrocyte density and concurrent increase in GFAP+ spatial coverage are detrimental or beneficial for the eye remains unknown. 

### 4.3. The RNFL and IPL Are Thinner in Older Myopic Marmoset Eyes

This study showed both RNFL and IPL thinning in all ages of myopic marmoset eyes studied, and the effect was more significant in the IPL of myopic eyes induced with myopia for longer. The thinning of the RNFL and IPL remained present after correcting for myopic magnification. Normal physiological aging without disease has been shown to exhibit ganglion cell inner plexiform layer (GCIPL) [116] and circumpapillary RNFL thinning [11]. Decreasing RGC density and RNFL thickness are both associated with the number of RGCs lost during normal aging, supporting the existence of a subset of age-dependent RNFL axons that are affected with age [116]. High myopia and glaucoma both show progressive GCIPL, RNFL, and ganglion cell complex [117] thinning. In fact, a thinning of the macular region has also been found to correlate with increasing degrees of myopia [118].

IPL thinning has been observed in eyes with early retinal ganglion cell injury [119] as well as in eyes diagnosed with advanced glaucoma [120], and the IPL’s structural alterations correspond to functional alterations in visual fields [121]. In our study, both the RNFL and IPL thinning remained significantly decreased after correcting for myopic magnification, confirming that myopic eye growth triggers a reorganization of the ganglion and supporting glial cell template that has been described in other eye diseases [107,122,123,124,125].

## 5. Conclusions

Our study confirms that the marmoset model can feasibly study cellular changes that may occur with myopia development and progression. This article aims to study the effect of sustained myopic defocus and eye growth on retinal astrocyte distribution and its relationship with inner retinal thicknesses. The results of this study show that experiencing myopia for longer exacerbates the decreased astrocyte density and increased GFAP+ spatial coverage seen in a progressive model of non-human primate myopia. To date, this study is the first to address sustained myopia’s effect on the retinal astrocyte template. However, the constructive or destructive implications of these changes, subsequent alterations to other parts of the neurovascular unit, and whether or not astrocyte function deteriorates with disease progression is unknown, further supporting the significance of this work. Future studies will quantitatively evaluate any functional alterations to the astrocyte template with both increasing age and increasing myopia.

## Figures and Tables

**Table 1:** Characteristics of control and myopic marmosets (axial length, refractive error, gender, age). Myopic marmosets initiated lens wear at 10 weeks old (72.0 ± 5.5 days) following an established protocol [34,37]. Daily morning contact lens insertion occurred between 8 and 10 am. Lights (700 lux) were turned on at 10 am after lenses were inserted, and subsequently removed 9 h later at lights off each day (9 h of light/15 h of dark). Contact lenses were either 3.6 or 3.8 mm base curve and 6.5 mm diameter, made of methafilcon A (55% water content, DK: 17), and fit 0.10 mm flatter than the flattest keratometry measurement. No corneal complications were observed in any of our treated myopic animals in this or earlier marmoset studies [34,36,37].

**6 m Control ID, Eye**	**Eye Length (mm)**	**Refraction (D)**	**Gender**	**Age (Days)**	**6 m Myope ID, Eye**	**Eye Length (mm)**	**Refraction (D)**	**Gender**	**Age (Days)**
C16, Right	10.259	−0.66	Female	268	B17, Right	10.900	−7.93	Female	214
C16, Left	10.241	−0.13	Female	268	B17, Left	10.894	−7.97	Female	214
G16, Left	10.279	−1.15	Male	215	O17, Right	10.492	−7.28	Male	204
H16, Right	10.286	−0.63	Female	205	O17, Left	10.212	−3.91	Male	204
H16, Left	10.307	−1.12	Female	205	P17, Right	10.554	−7.96	Female	183
					P17, Left	10.464	−3.08	Female	183
Average ± Standard Deviation	10.27 ± 0.03	−0.74 ± 0.4		232.2 ± 32.9	Average ± Standard Deviation	10.61 ± 0.3	−7.01 ± 1.8		200.3 ± 14.2
	*p* < 0.05	*p* < 0.01		*p* > 0.05					
**12 m Control ID, Eye**	**Eye Length (mm)**	**Refraction (D)**	**Gender**	**Age (Days)**	**12 m Myope ID, Eye**	**Eye Length (mm)**	**Refraction (D)**	**Gender**	**Age (Days)**
X15, Right	10.216	−1.12	Female	381	I19, Right	10.936	−7.34	Male	388
X15, Left	10.2202	−1.04	Female	381	J19, Right	10.791	−3.48	Male	388
S15, Right	10.181	−1.22	Female	396	J19, Left	10.766	−3.82	Male	388
Average ± Standard Deviation	10.20 ± 0.02	−1.12 ± 0.1		386 ± 8.7	Average ± Standard Deviation	10.83 ± 0.1	−4.08 ± 2.1		388 ± 0.0
	*p* < 0.05	*p* < 0.01		*p* > 0.05					

## Data Availability

The data presented in this study are available upon reasonable request from the corresponding author.
